# Barriers for general practitioners in post-stroke follow-up within a fragmented healthcare system in Sweden – a focus group study

**DOI:** 10.1080/13814788.2026.2728342

**Published:** 2026-09-15

**Authors:** Maria Wolf, Maria Flink, Axel C. Carlsson, Mia von Euler, Jan Hasselström

**Affiliations:** ^a^ Department of Neurobiology, Care Sciences and Society, Section of Family Medicine and Primary Care, Karolinska Institutet, Sweden; ^b^ Academic Primary Health Care Centre, Stockholm Region, Sweden; ^c^ Stockholm Research and Development Unit for Older Persons, Region Stockholm, Sweden; ^d^ Sophiahemmet University, Stockholm, Sweden

**Keywords:** Post-stroke follow-up, secondary prevention, primary care, healthcare organisation, medication adherence, general practitioners

## Abstract

**Background:**

Secondary medical prevention after stroke is effective and well evidenced, making long-term follow-up crucial. This follow-up is mainly made by general practitioners (GPs) in Sweden. Low target achievement and up to fourfold practice variation in dispensation of secondary preventive drugs between different primary care units in Region Stockholm has been described. Although several factors in individual practices have been observed to be associated with insufficient target achievements, the underlying reasons for this variation are unclear.

**Objectives:**

To explore the perspectives of GPs concerning secondary preventive practices after stroke to shed light on areas of improvement

**Methods:**

This qualitative study was based on eight focus group discussions with 49 GPs in Region Stockholm. They were strategically invited in relation to target achievement, and the interviews (45–55 min) were conducted using a semi-structured interview guide. The interviews were transcribed and analysed using qualitative content analysis.

**Results:**

There were two themes: “Multifaceted mission in an insufficiently organized healthcare system” and “The complexity of adherence”. There were six categories that encompassed shortcomings in the areas of: sufficient time for the patients, the overarching structure of the healthcare system, the internal structure of follow-ups, medical adherence, the responsibility for renewals of medication and tools for adherence.

**Conclusion:**

The perspectives of GPs revealed structural problems at different levels in the health care system as well as difficulties in handling medication adherence indicating a need for improvement. GPs touched on solutions including increasing the number of GPs, education and improved referrals from hospital to primary care.

## KEY MESSAGES

- GPs find post-stroke follow-up difficult because of heavy workload
- The healthcare system does not facilitate standardised follow-up of patients
- Medication adherence remains challenging due to unclear responsibility for the medication list and lack of tools to improve adherence

## Introduction

Globally, stroke is the second most common cause of death after cancer, which makes the need for secondary prevention high [1].

There is broad evidence that secondary preventive medications with antihypertensive agents, antiplatelet agents, and statins are crucial for reducing the risk of recurrence of stroke, cardiovascular morbidity, and limit premature death [1]. A meta-analysis from 2021 shows that patients’ average adherence to secondary preventive medications is 64% after stroke [2], with similar figures in Swedish primary care [3].

Long-term follow-up after stroke is mainly managed by general practitioners (GPs) in primary care [4]. A Swedish registry study, from the Stockholm Region, found the proportion of patients with stroke being dispensed recommended secondary preventive medication to vary between 25 and 100% among primary care centres (PCCs) [5]. A high proportion of statin dispensation was associated with a higher number of specialists in general medicine at the PCC and with a higher share of patients registered with a specific GP [3]. Factors associated with a lower level of dispensed statins were fragmented care and privately run PCCs [3].

Swedish national guidelines state that the target level for the proportion of patients with stroke receiving secondary preventive medications is 80%, and since 2018, structured follow-up in outpatient care 3–6 months after stroke has been included [5].

Little is known about the professional factors and GPs’ perspectives on medical secondary prevention among patients with stroke. In a broader sense, there seems to be uncertainty among GPs about many aspects of secondary cardiovascular prevention in older age, the existing guidelines, the GPs’ own role, patient factors, and the organisation of care [6].

In the absence of specific information on which factors determine practice variation in primary care in relation to stroke prevention, one may turn to what has been important when implementing new guidelines to guide the understanding [4]. In a recent systematic review, the most cited barriers to guideline implementation in the care of patients with chronic disease were organisational factors and healthcare professionals’ lack of knowledge and skills [7]. In addition to these factors, unsatisfactory adherence to recommended medications may also be explained by the complexity of patient situations and comorbidities [8].

This study aimed to explore GPs perspectives on secondary preventive practices after stroke, to shed light on possible areas of improvement.

## Methods

### Setting

The healthcare system in Sweden is organised by 21 self-governed regions under national laws and regulations. The Stockholm Region provides healthcare to approximately 2.4 million persons who can choose with which of the 240 PCCs, they want to register. PCCs may be run publicly or privately under the same regional terms and tax funded by the region [9]. The patient fee for a GP visit and the medication costs are subsidised by the government.

A consultation for chronic diseases in primary care is 20–30 min long. For some diseases like diabetes mellitus type 2 many PCCs uses electronical recall systems with a waiting list to ensure structured care, but it is up to the manager of each PCC to decide how to use it.

**Figure 1.**
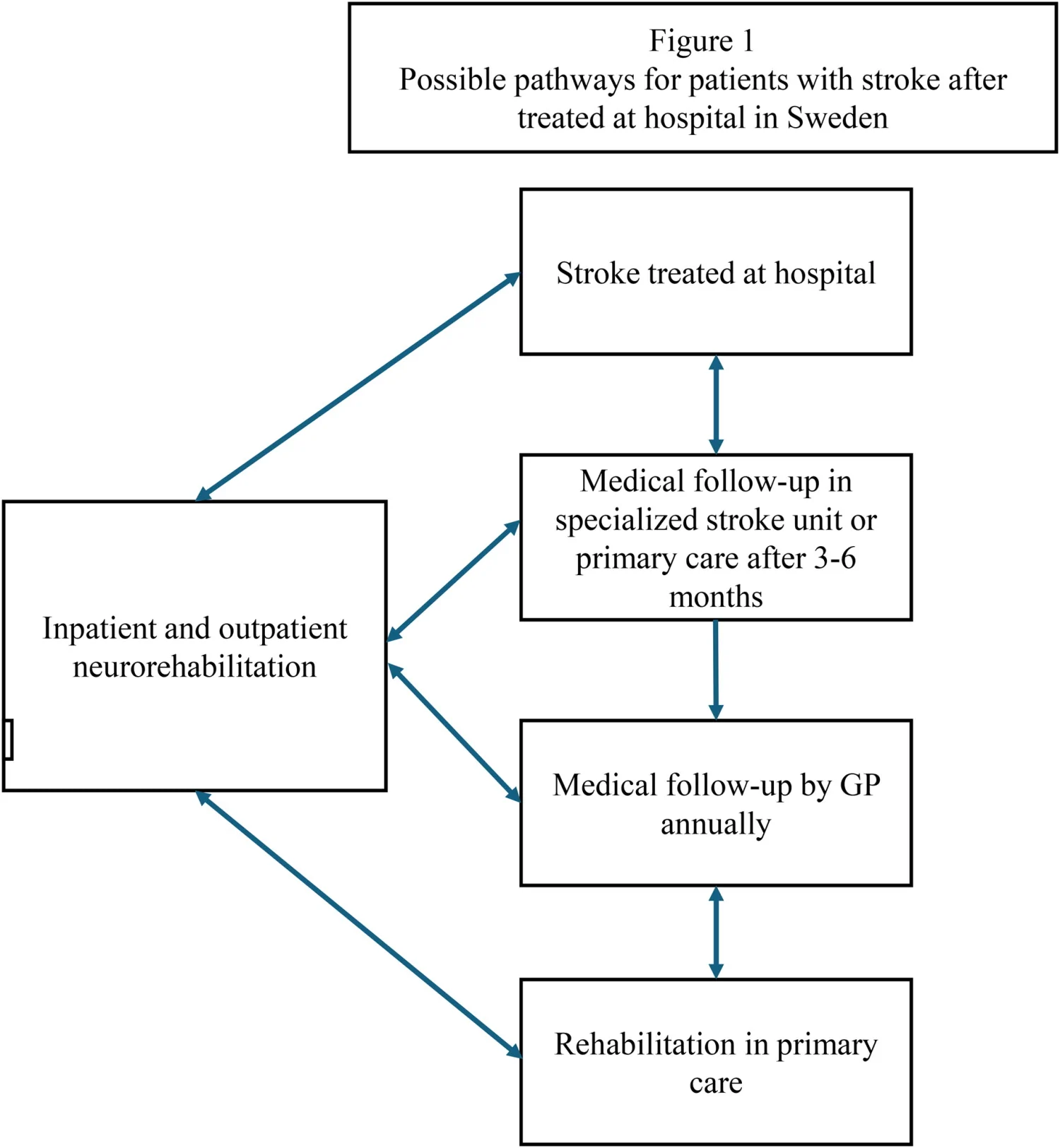
Possible pathways for patients with stroke after treated at hospital in Sweden. After treatment at hospital, patients may be sent to inpatient or outpatient rehabilitation. If rehabilitation is deemed not necessary patients may be sent directly for medical follow-up at a specialised stroke unit or primary care. At all stages new or unrecognised difficulties may necessitate rehabilitation at different levels.

Patients who are discharged to home after stroke treatment receive home rehabilitation by a specialised Neuro-team and a follow-up at a stroke unit or in primary care after 3–6 months. The responsibility for medical follow-up thereafter is referred to primary care. A schematic representation of the care pathways is shown in Figure 1.

### Study design

Physicians in eight different PCCs were invited to participate in focus group interviews. Nearly all physicians at each PCC participated ensuring a natural mix of different education levels in the focus groups. The physicians were defined as general practitioners (GPs) regardless of being a specialist in general medicine or a resident. A qualitative design was chosen to gain a deeper understanding of the GPs’ perspectives of the process of following up patients with a former stroke at the PCC in general, with a focus on the prescribing and treatment of secondary preventive medications according to national guidelines.

### Recruitment procedures

A purposive sampling of PCCs was used to ensure a variation in relation to size of the PCCs, whether publicly or privately run, the socioeconomic index, and whether PCCs were high or low in target achievement levels of stroke preventive medication. The GPs were recruited by email invitation to the operations manager of the PCC, who then invited the GPs to a lunch meeting. The GPs gave their written informed consent, and participant characteristics were collected before the interview.

### Data collection

In total, eight focus group interviews were conducted, assessed to hold adequate information power [10]. A moderator and a facilitator ran the interviews guided by a semi-structured interview guide (Supplemental file 1). The moderator was a GP undergoing doctoral studies (MW), and the facilitator (MF) was a medical social worker well experienced in qualitative studies. One interview was conducted at each PCC between December 2021 and May 2022. The participants in each group varied between five and nine persons, and the audiotaped interviews lasted between 45 and 55 min. The participants consisted of 25 men and 24 women aged 26 to 70 years and had been working 1–34 years after medical certification or between 1 and 28 years as a specialist in general medicine. Seven of the informants were residents and 42 were specialists in general medicine.

### Data analysis

The recorded interviews were transcribed without software assistance, analysed by qualitative content analysis [11]. The inductive analysis was framed in a constructivist perspective in which an iterative process of reading and analysing transcripts was applied. The analysis involved identifying, condensing and coding meaning units of the transcripts. Initially, MW and MF did one analysis each for each focus group session that was compared. After discussions and reflections of the coding, MW did the analyses under supervision involving iterative discussions, coding and re-reading of transcripts. The codes were clustered into sub-categories, categories and later themes based on thematic similarity during discussions between the authors. Findings were reported in accordance with the Standards for Reporting Qualitative Research [12]. Quotes from different GPs were chosen to illustrate the findings.

### Ethics

The study was conducted in accordance with good scientific practice with approval from the Swedish Ethical Review Authority. Ethical permit: 2021–04475.

## Results

The results were structured according to Table 1 in themes and categories.

**Table 1.** Themes and categories as a result of the analytical process.

Themes	Categories
Multifaceted mission in an insufficiently organised healthcare system	Time is a limiting factor
	Problems generated by shortcomings in the healthcare system
	Unstructured organisation
The complexity of medication adherence	Multiple factors influence adherence
	Unclear responsibilities for renewals
	Tools for adherence

### Theme 1: Multifaceted mission in an insufficiently organised healthcare system

#### Time is a limiting factor for the task

The lack of time for following up people with stroke and other chronic diseases was described as a main problem as well as the lack of appointments for patients due to too few GPs.

“The whole system is shaking, it has feet on clay, and it really doesn′t matter how fast we run”. (Female GP focus group 6)

The GPs found it difficult to accomplish everything according to the guidelines during one appointment, making the first follow-up after stroke a challenge. Sometimes the patients were not fully examined at the hospital and needed further referrals. Time constraints and high workload also limited discussions about their medical condition and medication, and it was difficult to arrange multiple appointments when needed. The GPs expressed insecurity about what they needed to follow up and what was done by the rehabilitation teams.

Following up patients with stroke could be time-consuming because of multiple illnesses and language difficulties. Sometimes complicated challenges due to patients’ fatigue, stress-related symptoms, and the need for assessing work capacity were time-consuming.

#### Problems generated by shortcomings in the healthcare system

The referrals from hospital physicians were perceived to vary greatly in their format and content. A description of what had been done at the hospital was sometimes missing, as well as the indications for newly introduced medications. The GPs were uncertain about whether follow-up was planned at the hospital stroke unit or meant to be in primary care. They expressed a need for a standardised template for referrals to ensure that no important information was left out. The templates would cover complete information about the medical condition, new medications with indications and recommended treatment duration, need for further rehabilitation and recommended time for follow-up. Simply referring to the discharge summary in the record was not sufficient according to the GPs, as they could not always find the relevant information there and thus had to spend too much time searching within scattered medical records.

“In the referral, there should be details about what happened, what was done, the medications prescribed, and suggestions for follow-up”. (Male GP 1 Focus group 1)

The GPs had noticed that there was an increased risk for the stroke diagnosis and follow-up being overlooked when patients changed physicians at the PCC. They believed that a long-term patient-physician relationship builds confidence and increases patients′ trust in physicians, making patients more receptive to new information, saving time for the GPs.

“I think things often go wrong when many different doctors are involved, changes in personnel may cause stroke follow-ups to be overlooked”. (Female GP 3 Focus group 4)

The GPs called for automated transfer of information from the hospital after treatment for stroke to the primary care record. As one of the hospitals in Stockholm had a different medical record system, the information transfer became complicated with risks of misjudgement and incorrect treatment.

The GPs experienced a gap in the professional relationship between themselves and other specialists. It was difficult to get in contact *via* telephone, and when GPs needed to refer a patient to a specialist, constant rejections of referrals and long waiting times created problems with associated delays.

Another described structural problem was the limited opportunity for the GPs to attend continuing education. It was often difficult to leave the PCC due to the shortage of GPs and fully booked schedules. There was great variation in whether local education was provided and how frequently it was organised at the PCC. Some of the GPs never had time allocated, while others had time set aside once a week. They generally thought they had not received enough continuing education about stroke.

#### Unstructured organisation

In lack of a cohesive common structure at the PCCs, following up patients after stroke was mainly considered the individual responsibility of the physicians. District nurses had either no or limited role in the follow-up of patients with stroke, unlike with patients with diabetes, who were followed up in a standardised manner.

There were also differences in the follow-up system comparing different PCCs. Some patients were scheduled for a follow-up appointment when a referral came from the hospital and then placed on a so-called waiting list (recall system) for annual check-up, while other patients were left to contact the PCC themselves. The first follow-up was conducted between six weeks and six months after the stroke, and there were different opinions about when the follow-up should be done. During the follow-ups, there also seemed to be variability in what was undertaken. Not all GPs regularly checked which medications the patients were using.

In general, the GPs were divided in their opinions on whether they should use a waiting list for patients with stroke or not. Some PCCs and some individual GPs used it, whereas others did not. Some GPs thought it was the patient′s own responsibility to book an appointment instead of using a waiting list, which could create a risk of a lack of available time slots when some patients missed their times.

“It is difficult with available appointments, yes, so patients themselves have to call and book a time. There are quite a few of them, and we can’t manage such a long waiting list.” (Male GP 1 focus group 5)

Other GPs thought it was very important for the patients having a waiting list.

“But this thing we were talking about with the waiting list, I do think it has a big impact on what signal we send out. If it ends up being like, ‘Well, it’s up to you to get in touch,’ or ‘We’ll book a follow-up in a year,’ there’s a big difference in what we’re signalling.” (Female GP 1 focus group 6)

### Theme 2: The complexity of medication adherence

#### Multiple factors influence adherence

The challenge with patients’ adherence to medications was perceived as a multifaceted issue, influenced by the patient’s personal circumstances, the effectiveness and side effects of the medication, as well as the level of support from relatives in the management of the medication process. There were different opinions among GPs about their own patients′ adherence. The sense was that it was most common for patients to refuse statins, but antihypertensive medication was also sometimes questioned. It was sometimes difficult to motivate patients to take medications because high blood lipids and high blood pressure generally do not present with symptoms. The GPs experienced that the patients′ knowledge of medications and reasons for adhering or not differed widely. For example, the GPs found that patients stopped taking medications when blood pressure and cholesterol levels were normal, that patients lacked medical information from the hospital, or that patients forgot about the stroke and therefore were lost to follow-up. Rumours about side effects could impact a patient′s motivation to follow their medication regimen.

“We have many patients, not just one, who say that statins are poison, not medicine”. (Male GP 1 focus group 5)

The GPs found the use of blister packs could help patients remember to take their medications. Explaining and motivating patients to adhere to their medication regime could be a pedagogical challenge, and some GPs thought they needed more training on this topic.

The GPs reflected on their own knowledge and values about secondary prevention, noting that they sometimes considered high blood sugar and high blood pressure more important to treat than high cholesterol. The time elapsed after the stroke incident was perceived as important and after some years other medical problems often took precedence, and the secondary preventive medication was forgotten. GPs also expressed the view that following up older patients with stroke was of less importance.

#### Unclear responsibility for renewals

The GPs perceived that the responsibility for medication management initially rested with the prescribing doctor at the hospital, but opinions differed on the responsibility after discharge from hospital. Some GPs believed that the referring doctor had full responsibility for the patient until the patient arrived at the PCC, while others thought that the PCC was responsible for the patient as soon as a referral was received.

Some GPs were very meticulous about reviewing the medication list from the record before renewing a prescription, while others renewed prescriptions without checking the indication for the medications. It was common for patients to call the PCC or submit a note for prescription renewals. Some GPs renewed prescriptions temporarily, often for three months at a time, while others renewed them for a whole year as the system in Sweden allows prescriptions with refills for at most one year. There were GPs, though, who did not renew prescriptions unless patients came for a follow-up visit, causing the patient to discontinue their medication until the next consultation. They expressed concerns that the lack of GPs and time for patient visits at the PCC was a system failure for which they could not compensate. Some primary care centres used a letter template in connection with requests for prescription renewals with a telephone number to call to book a new appointment.

“I haven’t worked here for that long, but from the patients I’ve met, there are quite a few who maybe haven’t really taken their medications as prescribed before. It could be that they ran out of medication or something, and they haven’t called in to get a renewal themselves.”(Male GP 1 focus group 4)

#### Tools for adherence

The GPs believed it was important to explain why patients should take secondary preventive medications to improve their adherence, and to discuss potential side effects and how to manage them. Some doctors noted that a patient-centred consultation model, focusing on the patient’s ideas, concerns and expectations worked well for discussing medications.

“It is important that the patient has the opportunity to express their concerns about medication and receive validation.” (Female GP 3 Focus group 7)

The GPs expressed that a concrete and effective way to handle side effects and patients’ fears of them was to titrate the medication, e.g. statins, starting from a very low dose with a gradual increase. However, not all GPs used this method. Some tried to motivate the patient that secondary prevention is like wearing a “seatbelt in traffic” or explaining the results of clinical studies. They noted that telephone follow-ups could be useful if a patient was concerned about side effects. One approach was to simplify the explanation of how elevated blood pressure and blood fats affect the blood vessels. It required extra educational effort to explain the importance of taking medications for high blood pressure and high cholesterol, which do not present symptoms. When writing prescriptions, GPs thought it was important to state the indication on the prescription so that the patient could see it on the label of the medication package. Some patients who experienced side effects or concerns about them needed more frequent follow-ups, which could be a challenge. The GPs requested training in motivational interviewing, which they had heard was effective for helping patients increase their motivation to take medication.

## Discussion

This qualitative study describing GPs’ experiences of secondary prevention for patients with stroke sheds light on why there may be deficiencies in the follow-up of patients with stroke. The overarching theme is that it is a difficult mission for GPs to follow up stroke according to guidelines, underpinned by two sub-themes: “multifaceted mission in an insufficient healthcare organization” and “the complexity of adherence”.

As in our study, time is often stated as a prime limiting factor for achieving a high quality of care in primary care [13]. In most Western health care systems, an increase in workload per primary care visit with more to do in less time has been described [14]. A problem with limited time for patients in primary care is that the chosen interventions and patient groups may be crowding out other patient groups [15]. Time seems to play an important role in primary care, as longer consultations improve health promotions, patient enablement, the quality of record keeping, and improved quality of life as well as patient enablement [13]. There is a public understanding of the shortage of GPs illustrated by the discrepancy between the Swedish National Board of Health’s recommended number of 1100 persons per GP and the actual double amount registered [16]. In Sweden, only 32 percent of the population are registered with a specific GP compared to an average of 81 percent in other OECD countries [17], yet most people in Sweden are registered with a PCC and they can change PCCs by a simple administrative measure.

Our study highlights that GPs experience a lack of information in referrals from secondary care. An enhanced preparation for discharge, information and support for self-management to patients post-discharge in the referral-based care transition after stroke have been called for [18]. Continuity of care was indirectly raised by our informants both from the perspective of provider continuity and information continuity, factors well-known to enhance medical results and reduce hospitalisations as well as being desired by patients [19]. A patient recall system with a waiting list was used by some of the GPs for patients with stroke. Although the evidence for patient recall systems overall is weak, it has been shown that regular check-ups are important for risk factor management among patients with diabetes [20]. Nurse-led telephone appointments have been shown to have positive effects on secondary preventive measures in patients with stroke [21], but this was not used among the PCCs in this study. A recent systematic review demonstrated that integrated care, incorporating elements touched upon in this study, like person-centredness, collaborating multiprofessional teams, psychological intervention and cardiovascular risk factor management, was associated with reductions in recurrent stroke, anxiety and depression and positive benefits on risk factors and quality of life [22].

A lack of built-in systems for continuing education in primary care for GPs is a reiterated theme also appearing in our study [4]. Swedish GP specialists lack the obligation to recertify their specialty, as is common in many other countries. When discussing efficacy, educational outreach visits with audit and feedback using GPs as academic detailers have been suggested to support and improve the quality of the GPs’ work [23].

The lack of an internal structure at the PCC was seen as a major obstacle by the GPs who struggled to follow guidelines. One possible reason patients are not properly followed up may be due to patients with stroke often being multimorbid [24]. Specifically, only 19% had strokes as the main topic, and the check-up of blood pressure, LDL, and lifestyle were insufficient when the treatment and follow-ups in primary care after stroke were reviewed [25]. Modern evidence-based suggestions on how to address multimorbidity include setting goals and planning for future care, deciding on the responsibility for coordination of care, communicating the plan and responsibility, agreement of timing for follow-up and review of medicines and other treatments [26]. All these issues were touched upon by the GPs in our study, simultaneously expressing limited ability to address them. On the other hand, some PCCs seemed to be able to handle the challenges, pointing to the importance of both the overall and local organisation of healthcare. The complexity of general practice, where even a serious condition loses the competition for attention to other important issues, calls for both individual efforts of GPs as well as systematic approaches of local and regional management. The present study describes local conditions in Sweden but studies in other countries have shown that follow-up care after stroke is sub-optimal indicating similar problems for patients with stroke [27,28].

Medication adherence, a complex process involving both physician and patient, emerged as an important issue in our investigation. While most patients are prescribed antihypertensives, antiplatelets, anticoagulants, and statins directly after having an ischaemic stroke, the persistent users of the drugs decline progressively during the first two years [5]. The reasons for non-adherence to medications could be many, and one main reason discussed by our informants was patients’ concerns about potential side effects [29]. In line with our study, the GPs′ own knowledge and attitudes about medication could also affect the work with patients′ adherence. GPs in our study expressed that patients with stroke were not prioritised as they were older and had several comorbidities. A systematic review shows that patients′ age may be a factor in the care they receive after an acute stroke. The possible influence of patients′ age on clinical decision making is complex and can sometimes be reasonable [30].

Interviews with patients identified two main barriers to adherence, patient level barriers and medication level barriers [31]. Factors predictive of non-adherence are patients’ concerns about treatment, lack of support, polypharmacy and having a more severe stroke [32]. A person-centred consultation where patients express their ideas, expectations and concerns, was one way to handle adherence [33], also expressed by the GPs in our study. Knowledge of other suggested tools for handling non-adherence seems to be lacking among many GPs [34]. The GPs thought that the law-regulated labelling of the medication on the packages is important for patients evidenced by a systematic review [35].

In our study, most GPs thought that they had the responsibility for renewals, but it was unclear for them when their responsibility started and to what extent this was the patient′s task. This varying perception of responsibility for the patients′ drug list and renewals [36] and healthcare providers seem to fluctuate between patients having all responsibility or having a shared responsibility for their medication adherence [37].

### Strength and limitations

A strength of this study is that the interviews were performed with GPs working at eight different primary care centres from different areas in Region Stockholm, including both PCCs with high and those with low achievement of medication targets. This contributes to credibility and transferability. The GPs had different lengths of working experience, also adding to quality. We took care to include the results of vigorous discussions of the varied experiences and perspectives of GPs, adding to high information power. Diverse author backgrounds gave varied perspectives during data analysis. A limitation is that it may be difficult to elicit individual, self-critical opinions from GPs in a focus group interview. As a qualitative study, this is hypothesis-generating and quantitative methods are needed to demonstrate if these findings are more generally held.

## Conclusion

This study adds knowledge on GP-related factors on secondary preventive practices after stroke. It describes difficulties with stroke follow-ups and medical adherence at two main levels, the healthcare organisational and the individual patient-GP-level. The lack of GP staffing causing general time constraints, limited common structure for following up patients with stroke, and GP difficulties in handling adherence deficiencies with patients are well illustrated in this study. GPs touched on solutions including increasing the number of GPs, education, and improved referrals from hospital to primary care. This highlights the need for re-organisation and prioritisation of primary care. Most likely, other groups of chronically ill patients would also benefit from a better-structured healthcare.

## Supplementary Material

Supplemental Material

Supplemental Material

Supplemental Material

## Data Availability

The data that supports the findings of this study are available on reasonable request from the corresponding author. The data is not publicly available due to privacy or ethical restrictions.
