# Supplementary material for: Barriers for general practitioners in post-stroke follow-up within a fragmented healthcare system in Sweden – a focus group study
Source: Eur J Gen Pract. 2026 Sep 15;32(1):2728342. doi: 10.1080/13814788.2026.2728342 (PMC13580359; doi:10.1080/13814788.2026.2728342)
Supplement: Supplementary file 1 [file IGEN_A_2728342_SM9965.docx]

Figure 1
Possible pathways for patients with stroke after treated at hospital in Sweden

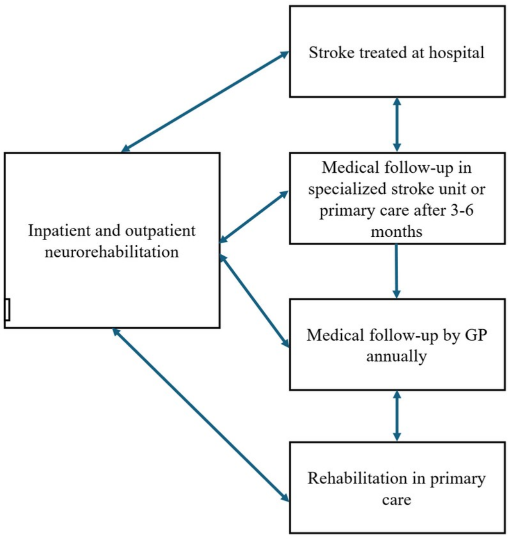

Legend to figure 1

After treatment at hospital, patients may be sent to inpatient or outpatient rehabilitation. If rehabilitation is deemed not necessary patients may be sent directly for medical follow-up at a specialized stroke unit or primary care. At all stages new or unrecognized difficulties may necessitate rehabilitation at different levels.
