# Supplementary material for: Barriers for general practitioners in post-stroke follow-up within a fragmented healthcare system in Sweden – a focus group study
Source: Eur J Gen Pract. 2026 Sep 15;32(1):2728342. doi: 10.1080/13814788.2026.2728342 (PMC13580359; doi:10.1080/13814788.2026.2728342)
Supplement: Supplementary file 1 [file IGEN_A_2728342_SM9964.docx]

Table 1. Themes and Categories as a result of the analytical process

| Themes | Categories |
| --- | --- |
| Multifaceted mission in an  insufficiently organized healthcare system | Time is a limiting factor |
|  | Problems generated by shortcomings in the healthcare system |
|  | Unstructured organisation |
| The complexity of medication adherence | Multiple factors influence adherence |
|  | Unclear responsibilities for renewals |
|  | Tools for adherence |
